# Associations between early experiences of thought interference and auditory-verbal hallucinations with first-rank symptoms and suicidality in adulthood

**DOI:** 10.1192/bjo.2024.784

**Published:** 2024-09-19

**Authors:** Hannah Gofton, Henrietta Rodriguez, Trinity Sheridan-Guest, Daniel H. Baker, Clara S. Humpston

**Affiliations:** Department of Psychology, University of York, York, UK; School of Psychology, University of Birmingham, Birmingham, UK

**Keywords:** ALSPAC, psychosis, hallucinations, thought interference, suicidality

## Abstract

**Background:**

Suicide is one of the major causes of premature death in patients diagnosed with a schizophrenia-spectrum psychotic disorder. However, associations between psychotic-like experiences in youth and suicidality in later life remain under-researched.

**Aims:**

We aimed to investigate any associations between early experiences of thought interference and auditory-verbal hallucinations (AVHs) with first-rank symptoms of schizophrenia and suicidal thoughts and behaviours in adulthood.

**Method:**

This study used data from the Avon Longitudinal Study of Parents and Children (ALSPAC). We calculated combined thought interference score at ages 11 years 8 months, 13 years 1 month, 14 years 1 month and 16 years 6 months. We also assessed AVHs at the same age points. For outcome variables, we used specific variables measuring delusions of control, AVHs and suicidality at 24 years of age. We carried out logistic regressions and mediation analyses to assess the relationships among these variables.

**Results:**

Thought interference and AVHs at all ages throughout childhood and adolescence were associated with suicidal thoughts and behaviours, and also with clinically more significant symptoms of delusions of control and AVHs at age 24. Substance use-induced psychotic-like experiences mediated a large proportion of the relationship between early psychotic-like experiences and suicidality in later life.

**Conclusions:**

Thought interference and AVHs in childhood and adolescence are associated with first-rank symptoms and suicidality in adulthood. Mental health interventions in children and adolescents need to take into account the impact of specific psychotic-like experiences and allow for the early detection of thought interference and AVH-related symptoms.

Although suicide is a leading cause of premature death in individuals diagnosed with schizophrenia, associations between psychotic-like experiences in youth and suicidality in later life remain under-researched. These may include psychotic-like experiences and symptoms that are extremely intrusive, such as auditory-verbal hallucinations (AVHs), delusions of thought interference (including thought insertion, withdrawal, broadcast and mind-reading experiences) and related phenomena, such as delusions of control and somatic passivity.^1^ Most of these are first-rank symptoms of schizophrenia. These intrusions into one's consciousness are, unsurprisingly, highly distressing and such distress may differ qualitatively and quantitatively from mood disturbances. Crucially, these symptoms directly breach the boundaries between the self and other within a person (i.e. ego-boundaries), leading to intense feelings of fear, dread, threat and psychological annihilation.

Previous research focusing on the associations between psychotic symptoms and suicidal thoughts and behaviours often does not differentiate between the contents of psychotic symptoms other than broad categories of delusions and hallucinations and relies heavily on questionnaires and/or self-reports. Experiencing psychotic symptoms at multiple points in life has been strongly associated with suicidal ideation and attempts. Connell et al^2^ used data from the Youth Self Report Questionnaire and/or Young Adult Self Report Questionnaire, which formed two time points, with participants aged 14 at baseline and followed up until age 21. Experiencing hallucinations at both ages increased the likelihood of suicidal ideation and behaviours compared to only reporting hallucinations at age 14. This study utilised measurements taken 7 years apart with minimal information surrounding the frequency or intensity of the hallucinations, and it is difficult to ascertain any persistence or reoccurrence of the reported hallucinations or their content.

Hielscher et al^3^ explored the correlation between AVH experiences during adolescence and the manifestation of suicidal thoughts and behaviours in adulthood. To evaluate this relationship, participants completed the Self-Harm Behaviour Questionnaire to gauge suicidal tendencies and The Diagnostic Interview Schedule for Children to assess AVH experiences. The study revealed that adolescents facing psychological distress and AVH experiences are more likely to engage in suicide attempts later in life.

Yates et al^4^ conducted a meta-analysis of 2540 studies to explore the clinical relevance of psychotic experiences as indicators of risk for subsequent suicidal behaviours. They demonstrated that individuals reporting psychotic experiences exhibited elevated odds of experiencing suicidal ideation, future suicide attempts, and future suicide death. The researchers concluded that these experiences serve as a significant clinical marker, highlighting an augmented risk for future suicidal thoughts and behaviours. In another meta-analysis, Huang et al^5^ found that psychosis significantly increased the likelihood that an individual would have suicidal thoughts and attempt suicide, particularly positive symptoms, which resulted in an increased risk of suicide. Based on these findings, they concluded that psychosis is a significant risk factor for suicide ideation, attempt and eventual death. In addition, the finding that positive symptoms particularly increased suicide risk provided support for the present investigation.

We analysed data from a large population-based birth cohort in the UK and examined the prevalence of thought interference and AVH experiences in children and adolescents aged 11–16. We aimed to investigate any associations between these early experiences and delusions of thought interference, AVHs and suicidal thoughts and behaviours aged 24. We also aimed to elucidate the potential mediating roles played by participant gender, psychotic-like experiences induced by substance use, those related to fever and sleep (e.g. hypnopompic and hypnagogic hallucinations) and distress associated with such experiences.

## Method

### Participants

This study used data from the Avon Longitudinal Study of Parents and Children (ALSPAC),^6–8^ which is a large population-based birth cohort. Pregnant women resident in Avon, UK, with expected dates of delivery between 1 April 1991 and 31 December 1992 were invited to take part in the study, and the initial number of pregnancies enrolled was 14 541. Of the initial pregnancies, there were 13 988 children who were alive at 1 year of age. The total sample size for analyses using any data collected after the age of seven is 15 447 pregnancies, of which 14 941 children were alive at 1 year of age. In total, 14 203 unique mothers were initially enrolled in the study, and as a result of additional recruitment, 14 833 unique women enrolled in ALSPAC as of September 2021. Ethical approval for the study was obtained from the ALSPAC Ethics and Law Committee and the Local Research Ethics Committees, and written informed consent for the use of data collected via questionnaires and clinics was obtained from participants following the recommendations of the ALSPAC Ethics and Law Committee at the time. Study data were collected and managed using REDCap (Research Electronic Data Capture) tools hosted at the University of Bristol.^9^ REDCap is a secure, web-based software platform designed to support data capture for research studies. Data analysis for the current study was conducted between 1 June 2023 and 29 February 2024. The study website contains details of all the data that is available through a fully searchable data dictionary and variable search tool: http://www.bristol.ac.uk/alspac/researchers/our-data/.

### Measures

Both experiences of thought interference and AVHs in childhood and adolescence (between ages 11 years 8 months and 16 years 6 months) were assessed by the following Child Completed Questionnaires: Watches and Funny Feelings (11 years 8 months), Reading and Singing (13 years 1 month), Life of a Teenager (14 years 1 month) and Life of a 16+ Teenager (16 years 6 months). These questionnaires enquired about unusual experiences relating to thought and perception, such as mind-reading, feelings of being under control of some special force or power and hearing a voice speaking to the young person when no one is present.

The outcome variables on psychotic-like experiences were derived from the Psychosis-Like Symptoms Interview (PLIKS), administered by a clinician, at age 24 according to criteria defined by the World Health Organization.^10^ PLIKS consists of 12 questions pertaining to delusions (persecution, reference, etc.), hallucinations and thought interference (withdrawal, insertion, broadcast). A psychotic-like experience is only scored as positive by the clinician if strongly suspected or definitely present. Psychotic-like experiences were assessed conservatively (‘rated down’) to avoid false positives or over-rating of otherwise nonclinical phenomena. As such, we have chosen to use the term ‘psychotic-like experience’ rather than ‘psychotic-like symptom’ or ‘psychotic experience’ to emphasise the fact that although these experiences might be clinically significant for some, they do not always cross the clinical threshold into a diagnosable psychotic disorder or cause functional impairment. Additional questions were asked regarding whether a psychotic-like experience was induced by substance use, whether it was a fever- or sleep-related phenomenon (e.g. hypnagogic or hypnopompic hallucinations) and whether such an experience caused any distress in the participant. Suicidal thoughts and behaviours at age 24 (self-harming with suicidal intent) variables were assessed by the Clinical Interview Schedule-Revised (CIS-R),^11^ with self-harm and suicide forming a separate set of questions within the CIS-R.

### Statistical analyses

Statistical analyses were conducted in R Studio running R version 4.3.2 for descriptive statistics, regression analyses and receiver-operating characteristic (ROC) curves and Stata/MP version 18 for Windows for mediation analyses. For predictor variables (psychotic-like experiences in childhood and adolescence), we calculated a combined thought interference score, which was defined as any combination of delusions or delusion-like experiences of mind reading, thought broadcast, thought echo, thought insertion and thought withdrawal at ages 11 years 8 months, 13 years 1 month, 14 years 1 month and 16 years 6 months. We also assessed AVHs, defined as hearing a voice speaking when no one is present, at the same ages. For outcome variables, we used specific variables measuring delusions of control, AVHs and suicidal behaviours (defined as a combination of suicidal thoughts and self-harming behaviours with suicidal intent) at 24 years of age. We recoded all variables into binary variables (1 = definitive or suspected yes, 0 = no to specific symptoms and experiences).

We compared individuals with and without psychotic-like experiences as children and adolescents using Pearson's chi-squared tests. We carried out logistic regression analyses and calculated odds ratios and 95% CIs between all predictor and outcome variables. The odds ratios represent the ratio of probabilities of suicidality at age 24 between people with and without psychotic-like experiences in childhood and adolescence. Both unadjusted models and models adjusted for gender, substance use-induced psychotic-like experiences, fever- and sleep-induced psychotic-like experiences and distress associated with psychotic-like experiences were performed. Using the regression coefficients, we also constructed ROC curves to demonstrate the specificity and sensitivity of each of the unadjusted models.

Two sets of mediation analyses were performed using the *khb* command in Stata, one using delusion of control at age 24 as the independent variable and the other using AVHs at age 24 as the independent variable. In both cases, suicidal thoughts and behaviours at age 24 were the dependent variable. Mediators in both sets of analyses were gender at birth, psychotic-like experiences induced by sleep and substance use and distress associated with psychopathology. Fever-induced psychotic-like experiences were not used as a mediator because available data for this variable were extremely limited. Direct, indirect and total effects, as well as the total proportion of effect mediated, were calculated with odds ratios and 95% CIs.

Missing data were excluded from the current study as we were unable to meet the assumptions of missing at random for multiple imputation; given the heavily stigmatised nature of such experiences and symptoms, as well as their potential impact on individuals’ functioning, missing data was unlikely to have occurred by chance.

## Results

### Sample characteristics

At age 24, the largest sample size was 3964 for suicidal thoughts and behaviours, followed by 3886 for AVHs and 3880 for delusions of control (Table 1, descriptive statistics). Delusions of control were reported by only 31 of the sample at age 24 (0.80%), whereas AVHs were reported by 412 individuals (10.6%). Some 1156 individuals reported yes to suicidal thoughts and behaviours in their lifetime at age 24 (29.2%). From available data, 16 (51.6%) males reported delusions of control at age 24, 182 (44.0%) males reported AVHs and 422 (36.5%) males reported lifetime suicidal thoughts and behaviours. Of all participants, 46 met the criteria for a psychotic disorder at age 18, whereas only 3 had ever received a formal diagnosis of schizophrenia.

**Table 1:** Descriptive statistics and psychopathology variables in relation to suicidal thoughts and behaviours

Variable	Delusions of control at 24 years of age			Auditory-verbal hallucinations at 24 years of age			Suicidal thoughts and behaviours at 24 years of age		
	Yes (*N* = 31)	No (*N* = 3849)	*P*-value	Yes (*N* = 412)	No (*N* = 3474)	*P*-value	Yes (*N* = 1156)	No (*N* = 2808)	*P*-value
Gender (%)									
Male	16 (51.6)	1437 (37.3)	<0.001	182 (44.0)	1275 (36.7)	<0.001	422 (36.5)	1057 (37.6)	<0.001
Female	15 (48.4)	2412 (62.6)		230 (56.0)	2199 (63.3)		734 (63.4)	1749 (62.3)	
Delusions of thought interference (%)^a^									
11 years 8 months	10 (32.3)	1216 (31.6)	<0.001	137 (33.3)	1090 (31.4)	<0.001	414 (35.8)	843 (30.0)	<0.001
13 years 1 month	7 (22.6)	529 (13.7)	<0.001	86 (20.9)	451 (13.0)	<0.001	206 (17.8)	342 (12.2)	<0.001
14 years 1 month	8 (25.8)	365 (9.50)	<0.001	70 (17.0)	304 (8.75)	<0.001	164 (14.2)	219 (7.80)	<0.001
16 years 6 months	*7* (*19.4*)	607 (11.3)	<0.001	115 (28.0)	500 (14.4)	<0.001	284 (15.9)	346 (12.3)	<0.001
Auditory-verbal hallucinations (%)									
11 years 8 months	10 (32.3)	824 (21.4)	<0.001	129 (31.4)	705 (20.3)	<0.001	322 (27.8)	527 (18.8)	<0.001
13 years 1 month	9 (29.0)	555 (14.4)	<0.001	121 (29.4)	443 (12.8)	<0.001	231 (20.0)	340 (12.1)	<0.001
14 years 1 month	9 (29.0)	389 (10.1)	<0.001	110 (26.8)	290 (8.35)	<0.001	189 (16.3)	216 (7.69)	<0.001
16 years 6 months	–	–	–	84 (20.4)	176 (5.07)	<0.001	137 (11.8)	134 (4.77)	<0.001

a. Defined as any combination of delusions of mind reading, thought broadcast, thought echo, thought insertion and thought withdrawal. Cell counts smaller than 5 have been removed owing to Avon Longitudinal Study of Parents and Children reporting requirements.

### Associations between psychotic-like experiences in childhood and adolescence and those in adulthood

Table 2 shows the unadjusted associations between delusions of thought interference and AVHs in childhood/adolescence (ages 11–16) and delusions of control and AVHs in adulthood (age 24). Experiences of thought interference at age 14 were associated with the presence of delusions of control at age 24 (odds ratio = 3.90, 95% CI 1.55–9.16, *P* = 0.002), whereas experiences of thought interference at ages 13, 14 and 16 were associated with the presence of AVHs at age 24 (odds ratios 2.10–3.22, 95% CIs 1.59–4.34, *P*s < 0.001). AVHs at all ages throughout childhood and adolescence except age 16 were associated with the presence of delusions of control at age 24 (odds ratios 3.05–4.35, 95% CIs 1.22–10.2, *P*s 0.001–0.016), and AVHs at all ages throughout childhood and adolescence were associated with AVHs at age 24 (odds ratios 2.10–6.68, 95% CIs 1.59–9.04, *P*s < 0.001). However, these effects diminished after controlling for confounders (Supplementary Tables 1 and 2 available at https://doi.org/10.1192/bjo.2024.784), and some regression models did not converge because of the very small sample size for some of the variables.

**Table 2:** Unadjusted associations between delusions of thought interference and auditory-verbal hallucinations in childhood and psychopathology outcomes at 24 years of age

Variable	Delusions of control at 24 years of age			Auditory-verbal hallucinations at 24 years of age			Suicidal thoughts and behaviours at 24 years of age		
	*β*	*P*-value	Odds ratio (95% CI)	*β*	*P*-value	Odds ratio (95% CI)	*β*	*P*-value	Odds ratio (95% CI)
Delusions of thought interference^a^									
11 years 8 months	0.435	0.333	1.54 (0.63–3.78)	0.280	0.022	1.32 (1.04–1.68)	0.289	<0.001	1.33 (1.14–1.56)
13 years 1 month	0.815	0.077	2.26 (0.85–5.39)	0.740	<0.001	2.10 (1.59–2.74)	0.492	<0.001	1.64 (1.35–1.98)
14 years 1 month	1.360	0.002	3.90 (1.55–9.16)	0.948	<0.001	2.58 (1.91–3.46)	0.733	<0.001	2.08 (1.67–2.59)
16 years 6 months	0.791	0.104	2.21 (0.81–5.63)	1.201	<0.001	3.22 (2.54–4.34)	0.928	<0.001	2.55 (2.12–3.07)
Auditory-verbal hallucinations									
11 years 8 months	1.114	0.016	3.05 (1.22–7.70)	0.785	<0.001	2.19 (1.72–2.79)	0.548	<0.001	1.73 (1.46–2.04)
13 years 1 month	1.143	0.009	3.14 (1.29–7.31)	0.740	<0.001	2.10 (1.59–2.74)	0.653	<0.001	1.92 (1.59–2.32)
14 years 1 month	1.471	0.001	4.35 (1.79–10.2)	1.670	<0.001	5.31 (4.05–6.96)	0.935	<0.001	2.55 (2.05–3.16)
16 years 6 months	1.075	0.062	2.93 (0.82–8.36)	1.899	<0.001	6.68 (4.91–9.04)	1.043	<0.001	2.84 (2.20–3.66)

a. Defined as any combination of delusions of mind reading, thought broadcast, thought echo, thought insertion and thought withdrawal.

### Associations between psychotic-like experiences in childhood and adolescence and suicidality in adulthood

Also in Table 2, it can be seen that experiences of thought interference at all ages throughout childhood and adolescence were associated with suicidal thoughts and behaviours at age 24 (odds ratios 1.33–2.55, 95% CIs 1.14–3.07, *P*s < 0.001). Similarly, AVHs at all ages throughout childhood and adolescence were also associated with suicidal thoughts and behaviours at age 24 (odds ratios 1.73–2.84, 95% CIs 1.46–3.66, *P*s < 0.001). These effects diminished after adjusting for confounders (Supplementary Tables 1 and 2), and some regression models did not converge.

### Mediation analyses

Results for the mediation analyses are displayed in Table 3a and 3b. From Table 3a, substance use-induced psychotic-like experiences mediated 21.79% of the total effect (indirect effect odds ratio = 1.44, 95% CI 1.26–1.64, *P* < 0.001) between delusion of control and suicidal thoughts and behaviours at age 24, whereas Table 3b shows similar results for substance use-induced psychotic-like experiences (indirect effect odds ratio = 1.61, 95% CI 1.25–2.07, *P* < 0.001, percentage mediated = 40.87%) between AVHs and suicidality. The combined mediation effects from gender, sleep-induced psychotic-like experiences, substance use-induced psychotic-like experiences and distress associated with psychotic-like experiences mediated 60.67% of the total effect between AVHs and suicidality at age 24 (indirect effect odds ratio = 0.83, 95% CI 0.50–1.41, *P* = 0.504), yet the same model did not converge when using distress as a mediator for the association between delusion of control and suicidality, again because of a very small sample size for distress (positive *N* = 14).

**Table 3a:** Mediation analyses between delusion of control and suicidal thoughts and behaviours at age 24. Mediators: gender at birth, psychotic-like experiences induced by sleep and substance use and distress associated with psychopathology

Delusion of control	Suicidal thoughts and behaviours
	Odds ratio (95% CI)
**Gender**	
Total	5.20 (2.44–11.07), *P* < 0.001
Direct	5.24 (2.46–11.17), *P* < 0.001
Indirect	0.99 (0.97–1.01), *P* = 0.445
	**−0.56%**
**Sleep-induced psychotic-like experiences**	
Total	3.33 (0.69–16.01), *P* = 0.133
Direct	3.31 (0.69–15.94), *P* = 0.135
Indirect	1.00 (0.95–1.05), *P* = 0.850
	**0.38%**
**Substance use-induced psychotic-like experiences**	
Total	5.31 (2.42–11.67), *P* < 0.001
Direct	3.69 (1.68–8.12), *P* = 0.001
Indirect	1.44 (1.26–1.64), *P* < 0.001
	**21.79%**
**Distress associated with psychotic-like experiences**	
Total	Model did not converge – positive *N* = 14
Direct	
Indirect

**Table 3b:** Mediation analyses between auditory hallucinations and suicidal thoughts and behaviours at age 24. Mediators: gender at birth, psychotic-like experiences induced by sleep and substance use and distress associated with psychopathology

Auditory hallucinations	Suicidal thoughts and behaviours
	Odds ratio (95% CI)
**Gender**	
Total	3.17 (2.57–3.91), *P* < 0.001
Direct	3.19 (2.59–3.94), *P* < 0.001
Indirect	0.99 (0.98–1.01), *P* = 0.279
	**−0.54%**
**Sleep-induced psychotic-like experiences**	
Total	0.75 (0.13–4.18), *P* = 0.742
Direct	0.81 (0.14–4.53), *P* = 0.806
Indirect	0.93 (0.76–1.14), *P* = 0.467
	**25.01%**
**Substance use-induced psychotic-like experiences**	
Total	3.18 (2.57–3.94), *P* < 0.001
Direct	1.98 (1.43–2.76), *P* < 0.001
Indirect	1.61 (1.25–2.07), *P* < 0.001
	**40.87%**
**Distress associated with psychotic-like experiences**	
Total	0.57 (0.05–6.65), *P* = 0.652
Direct	0.67 (0.06–8.07), *P* = 0.765
Indirect	0.82 (0.50–1.35), *P* = 0.443
	**33.90%**
**Gender + Sleep + Substance + Distress**	
Total	0.74 (0.07–8.75), *P* = 0.816
Direct	0.89 (0.08–10.56), *P* = 0.927
Indirect	0.83 (0.50–1.41), *P* = 0.504
	**60.67%**

### Sensitivity and specificity

ROC curves (see Supplementary Figure 1) between each of the predictors (thought interference and AVHs in childhood and adolescence) and each of the outcome variables (delusions of control, AVHs and suicidality at age 24) were generated. For each ROC, sensitivity and specificity improved with the course of time, with age 16 having both the highest sensitivity (80%) and specificity (75%) for all predictor variables on all outcome variables.

## Discussion

We aimed to investigate the relationships between specific psychotic-like experiences in childhood and adolescence and suicidality in adulthood. Our main findings were that (1) experiences of thought interference and AVHs at all ages throughout childhood and adolescence were associated with suicidal thoughts and behaviours at age 24, and (2) the same experiences in childhood and adolescence were associated with clinically more significant symptoms of delusions of control and AVHs later at age 24, with the highest levels of sensitivity and specificity coming from age 16. Substance use-induced psychotic-like experiences mediated a large proportion of the relationship between early psychotic-like experiences and suicidality in later life.

These findings corroborate with previous research, such as Hielscher et al's study,^3^ supporting the notion that there is a relationship between positive symptoms of schizophrenia and suicidal tendencies. However, this relationship diminished after controlling for confounding factors, suggesting that the relationship could also be at least partially explained by other factors, including substance use-induced psychotic-like experiences. Nevertheless, it is clear that early experiences specifically resembling those in a schizophrenia-spectrum psychosis, rather than other more broadly defined psychotic-like experiences, are potentially predictive of more severe difficulties later in life, including suicidal thoughts and behaviours. The finding that psychotic-like experiences that involve a breach of a person's ego-boundary and impede one's sense of agency are specifically associated with suicidality is perhaps not surprising, yet represents a missed opportunity in many clinical encounters.

Substance-induced psychotic-like experiences, rather than distress associated with psychotic-like experiences, mediated the highest proportion of the relationship between thought interference and suicidality. In fact, only 14 individuals reported any distress resulting from their thought interference. The same pattern was not seen with AVHs, where distress alone mediated over 30% of the total effect. It may indicate that substance use-induced delusional thoughts, and perhaps not substance use by itself, have the highest impact on the risk to oneself,^12,13^ whereas for AVHs, the distress alone can lead to suicidal thoughts and behaviours. Indeed, previous research supports the idea that substance-induced delusional states may carry a higher risk to self even though the individual may not be overtly distressed in the same way that AVHs can cause distress, although the current evidence is inconlusive.^14,15^ It may be that delusions become more action-guiding when they are induced by substances, whereas AVHs have a more direct relationship with suicidality.

However, an alternative explanation could lie within how distress is assessed. In contrast with substance use-induced psychotic-like experiences where dichotomous answers may be readily adopted, the assessment of distress often loses its nuances in large-scale population surveys. Fialko et al's study^16^ suggested that the content, type and duration or severity of distress evoked by a delusion was impactful on the association between delusions and suicidal ideation. The dichotomous decision to code specific variables as present or not present may reduce the dimensions that delusions and associated distress are recorded under and limit the ability of this study to investigate these effects. In addition, their study found the strongest association between the number of delusions and the intensity of distress, which may have been difficult to investigate in the ALSPAC cohort, leading to limitations with sample size in participants reporting distress associated with thought interference.

It is also possible that the *kinds* of distress associated with psychotic-like experiences may be fundamentally different from those induced by depression or anxiety. Although the prevalence of suicidal thoughts and behaviours in depression may be higher than that in schizophrenia,^17,18^ suicidality in the latter diagnosis is frequently less predictable and potentially also more violent in its methods, which could lead to greater harm and threat to the patient's life. As a result, instruments used to assess distress in depression and anxiety may bear little value in capturing the kinds of distress in psychotic disorders. Nevertheless, we did not enter mood disturbance variables (as opposed to measures of psychological distress) into our model. It could be that it is not the psychotic-like experiences *per se* that are conferring risk, but rather other measures that are not reported in the current study. Indeed, there is evidence suggesting that psychotic-like experiences are a marker of severity of mental distress in general, rather than specific risk for psychosis.^19^ Although this is clearly a limitation, our aims were not focused on predicting psychosis risk based solely on early experiences of specific psychotic-like phenomena *or* suicidality, but rather to ascertain the relationships between these variables without necessarily inferring or asserting directions of causality. Future research using similar methods could contrast suicidality arising from depression-related mood disturbances with that from distress caused by psychotic-like experiences and psychotic disorders.

The finding that early experiences resembling first-rank symptoms in particular are associated with suicidality even 10 years later is in itself very telling. Although the clinical utility of first-rank symptoms for the differential diagnosis of schizophrenia has been brought under debate more recently,^20,21^ given the associations between early experiences resembling first-rank symptoms and risk to self as demonstrated by the current study, we ought to take these specific psychotic-like experiences more seriously for the purposes of suicide risk management at a minimum.

Most previous studies of this kind have focused on the relationships between *psychosis* more broadly and suicidality, instead of symptoms and experiences potentially more indicative of *schizophrenia*, the most severe form of psychotic disorders. Recent evidence suggests that first-rank symptoms can be conceptualised as ‘end-points’ of disturbances in a person's sense of self,^22,23^ which directly affect how external reality is perceived and how an individual navigates one's environment as well as relations with others. Even though self-disturbances are not assessed in the ALSPAC data-set, it would not be surprising that, given the close relationship between self-disturbances and first-rank symptoms, the former could be a generative factor towards suicidality in a way specific to schizophrenia as opposed to other psychoses.

Nevertheless, not all first-rank symptoms are used in the current analysis. For example, the AVH variables do not differentiate between second- and third-person hallucinations, or any phenomenological features of AVHs (commenting, commanding, etc.), which could mean that some specificity is likely to have been lost. An additional difficulty with analysing this cohort is the already very small number of individuals experiencing, for example, command hallucinations that, despite their close relationships with delusions of control and somatic passivity, would not have allowed for any meaningful analytic approaches. This is similarly reflected in the small number (*N* = 31) of individuals reporting narrowly defined delusions of control at age 24. Again, this should not be surprising as the ALSPAC originates from the general population where first-rank symptoms are rare by definition. Ideally, one would restrict the analyses to those with psychotic-like experiences and suicidality in a subgroup of participants; however, this was not possible because of limitations in sample size. However, as all our variables are binary, the odds ratios obtained here should at least give some indication of the probabilities of suicidality in adulthood *given* the presence of early psychotic-like experiences. Other potentially important factors, including cognitive profiles and social deprivation, could also have played a role in both the development of psychotic-like experiences and suicidality, although cognition and social influences were not part of the research questions in the current study.

A final caveat when interpreting these findings is that, admittedly, the relationship between psychotic-like experiences and an eventual diagnosis of schizophrenia is weak at best for most individuals who may have been deemed at risk of developing a psychotic illness. Experiences of thought interference do not always lead to a delusional elaboration, and it is usually the latter that confers clinical status. Similarly, measures of suicidality (thoughts and behaviours) usually only show a limited relationship with actual suicide attempts, even though in the present study we intentionally omitted self-harm *without* suicidal intent in our analyses. The fact that only three participants in the ALSPAC cohort have ever received a diagnosis of schizophrenia specifically indicates that these observational findings may not necessarily predict eventual psychiatric diagnoses of a severe mental illness or any mental illness at all.^24^ This could have been reflected by the differences in predictive power at various ages, which could mean that these measures of psychotic-like experiences are not directly related to an emerging psychotic disorder. However, an added novel aspect of the current study is the use of specific psychotic-like experiences akin to first-rank symptoms of schizophrenia, rather than more broadly defined psychotic-like experiences.

### Clinical implications

Compared with common mental disorders such as depression and anxiety, suicidal thoughts and behaviours in schizophrenia are relatively under-researched areas, which can have serious impacts on clinical practice. Experiences of thought interference and AVHs can be extremely frightening for a young person and can force them to withdraw from their peers, which will likely worsen the outcome.^25,26^ The way in which most clinicians assess AVHs is also problematic, as in many cases AVH experiences are more akin to delusional convictions than true perceptual events.^27–29^ However, if identified accurately at the earliest point, AVHs and thought interference can be effectively managed in a young person. Our findings indicate that the first step is to enquire about such experiences; the purpose of early detection is not to simply jump to conclusions about future risk, but rather to engage in meaningful dialogues with young people during critical periods of development.

## Supporting information

Gofton et al. supplementary material 1 Gofton et al. supplementary material

Gofton et al. supplementary material 2 Gofton et al. supplementary material

Gofton et al. supplementary material 3 Gofton et al. supplementary material

## Data Availability

The data that support the findings of this study are available on request from https://www.bristol.ac.uk/alspac/ upon submission of formal data access proposals. The data are not publicly available owing to their containing information that could compromise the privacy of research participants.
