# Supplementary figures and images for: Associations between early experiences of thought interference and auditory-verbal hallucinations with first-rank symptoms and suicidality in adulthood
Source: BJPsych Open. 2024 Sep 19;10(5):e157. doi: 10.1192/bjo.2024.784 (PMC11457199; doi:10.1192/bjo.2024.784)

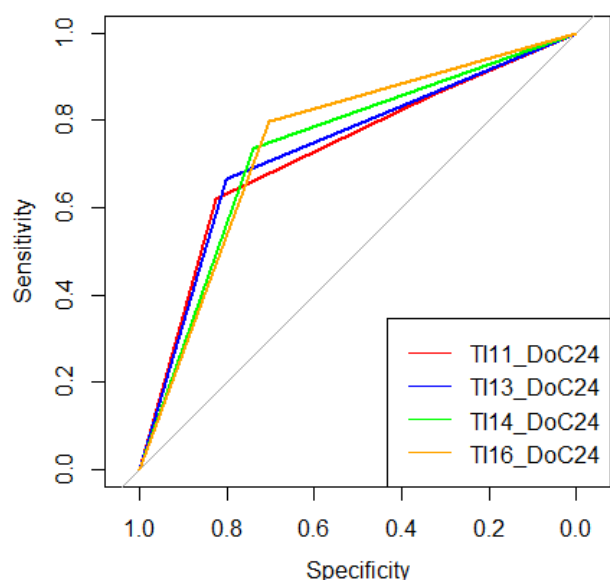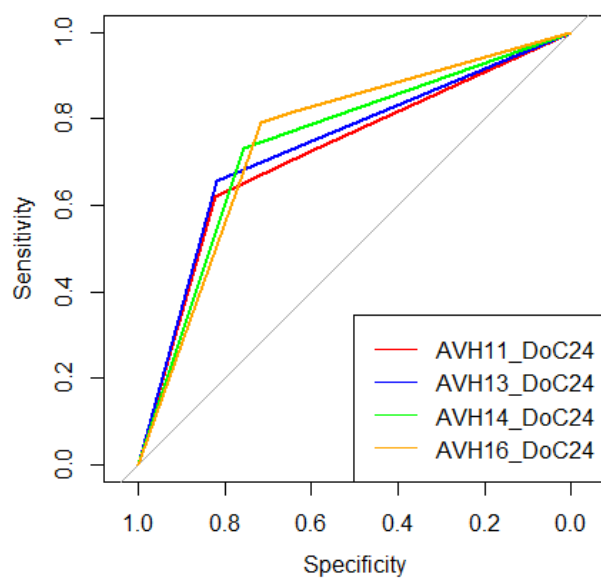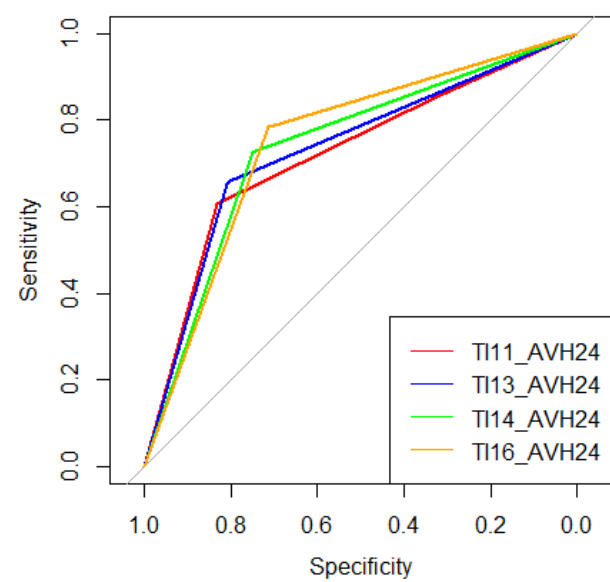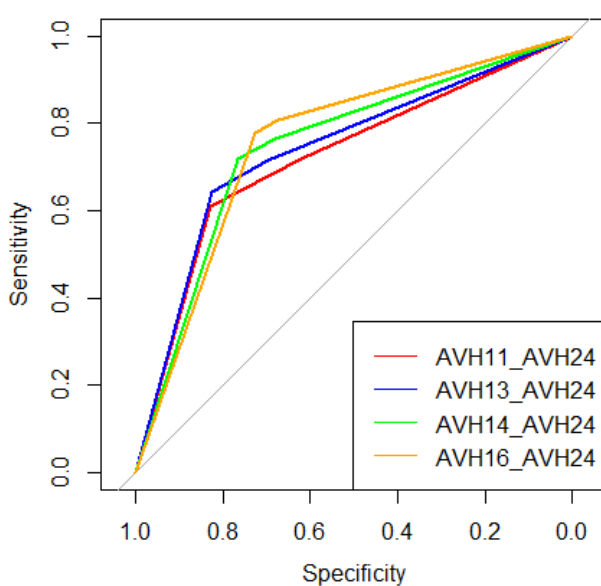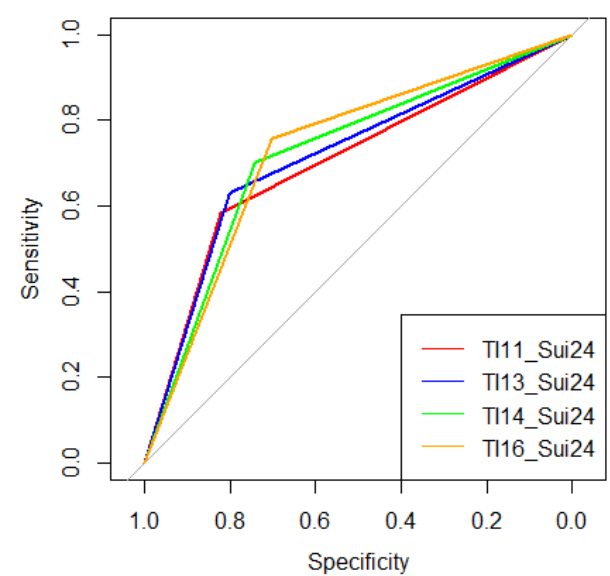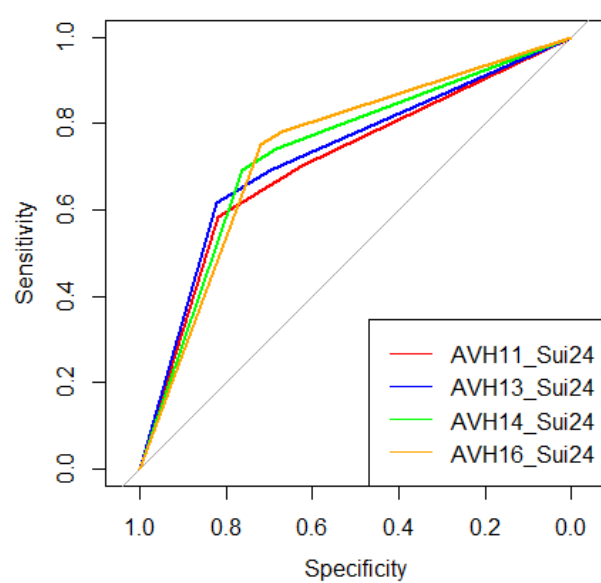

Supplement: Gofton et al. supplementary material 1 — Gofton et al. supplementary material [file S2056472424007841sup001.pdf]
