# Supplementary material for: Associations between early experiences of thought interference and auditory-verbal hallucinations with first-rank symptoms and suicidality in adulthood
Source: BJPsych Open. 2024 Sep 19;10(5):e157. doi: 10.1192/bjo.2024.784 (PMC11457199; doi:10.1192/bjo.2024.784)
Supplement: Gofton et al. supplementary material 2 — Gofton et al. supplementary material [file S2056472424007841sup002.docx]

**Supplementary Table 1.** Associations between delusions of thought interference, auditory-verbal hallucinations in childhood and psychopathology outcomes at 24 years of age, adjusted for sex at birth and experiences induced by sleep, fever and substance use. OR, odds ratio; CI, confidence interval.

| Variable | Delusions of control at 24 years of age | | | Auditory-verbal hallucinations at 24 years of age | | | Suicidal thoughts and behaviours at 24 years of age | | |
| --- | --- | --- | --- | --- | --- | --- | --- | --- | --- |
|  | β | P value | OR (95% CI) | β | P value | OR (95% CI) | β | P value | OR (95% CI) |
| Delusions of thought  Interference* |  |  |  |  |  |  |  |  |  |
| 11y 8m | -1.354 | 0.252 | 0.26(0.01-1.88) | 0.031 | 0.944 | 1.03(0.43-2.46) | 0.349 | 0.414 | 1.42(0.62-3.31) |
| 13y 1m |  | Non-convergence |  | 0.526 | 0.252 | 1.69(0.68-4.18) | 0.319 | 0.499 | 1.38(0.56-3.56) |
| 14y 1m | 1.200 | 0.186 | 3.32(0.53-21.6) | 0.298 | 0.560 | 1.35(0.48-3.63) | 1.165 | 0.046 | 3.21(1.10-11.0) |
| 16y 6m | 0.11 | 0.910 | 1.11(0.13-8.36) | 0.785 | 0.107 | 2.19(0.85-5.73) | 0.915 | 0.053 | 2.50(1.02-6.41) |
| Auditory-verbal  Hallucinations |  |  |  |  |  |  |  |  |  |
| 11y 8m | -0.174 | 0.805 | 0.84(0.19-3.31) | 0.900 | 0.490 | 2.45(0.23-70.1) | 0.283 | 0.363 | 1.33(0.72-2.45) |
| 13y 1m | 0.319 | 0.628 | 0.73(0.18-2.59) |  | Non-convergence |  | -0.006 | 0.983 | 0.99 (0.54-1.83) |
| 14y 1m | 0.225 | 0.737 | 1.25(0.33-4.84) |  | Non-convergence |  | 0.404 | 0.225 | 1.50(0.78-2.89) |
| 16y 6m | -0.217 | 0.803 | 0.81(0.12-4.16) |  | Non-convergence |  | 0.632 | 0.080 | 1.89(0.94-3.84) |
| *Defined as any combination of delusions of mind reading, thought broadcast, thought echo, thought insertion and thought withdrawal. | | | | | | | | | |
