# Supplementary material for: Associations between early experiences of thought interference and auditory-verbal hallucinations with first-rank symptoms and suicidality in adulthood
Source: BJPsych Open. 2024 Sep 19;10(5):e157. doi: 10.1192/bjo.2024.784 (PMC11457199; doi:10.1192/bjo.2024.784)
Supplement: Gofton et al. supplementary material 3 — Gofton et al. supplementary material [file S2056472424007841sup003.docx]

**Supplementary Table 2.** Associations between delusions of thought interference, auditory-verbal hallucinations in childhood and psychopathology outcomes at 24 years of age, adjusted for sex at birth, experiences induced by sleep, fever and substance use, and distress associated with psychopathology. OR, odds ratio; CI, confidence interval.

| Variable | Delusions of control at 24 years of age | | | Auditory-verbal hallucinations at 24 years of age | | | Suicidal thoughts and behaviours at 24 years of age | | | | |
| --- | --- | --- | --- | --- | --- | --- | --- | --- | --- | --- | --- |
|  | β | P value | OR (95% CI) | β | P value | OR (95% CI) | β | P value | | OR (95% CI) | |
| Delusions of thought  Interference* |  |  |  |  |  |  |  | |  | |  |
| 11y 8m | -1.297 | 0.272 | 0.27(0.01-2.01) | 0.055 | 0.902 | 1.06(0.44-2.54) | 0.376 | | 0.382 | | 1.46(0.63-3.42) |
| 13y 1m |  | Non-convergence |  | 0.527 | 0.254 | 1.69(0.68-4.18) | 0.333 | | 0.483 | | 1.39(0.56-3.62) |
| 14y 1m | 1.247 | 0.184 | 3.48(0.52-24.5) | 0.298 | 0.560 | 1.35(0.48-3.63) | 1.165 | | 0.046 | | 3.21(1.10-11.0) |
| 16y 6m | 0.095 | 0.929 | 1.10(0.11-9.50) | 0.781 | 0.109 | 2.18(0.85-5.73) | 0.909 | | 0.056 | | 2.48(1.01-6.40) |
| Auditory-verbal  Hallucinations |  |  |  |  |  |  |  | |  | |  |
| 11y 8m | -0.181 | 0.798 | 0.83(0.19-3.31) | 0.873 | 0.508 | 2.39(0.21-70.4) | 0.292 | | 0.353 | | 1.34(0.72-2.48) |
| 13y 1m | -0.332 | 0.615 | 0.72(0.18-2.56) |  | Non-convergence |  | -0.035 | | 0.910 | | 0.97(0.52-1.78) |
| 14y 1m | 0.212 | 0.753 | 0.12(0.32-4.78) |  | Non-convergence |  | 0.373 | | 0.266 | | 1.45(0.76-2.81) |
| 16y 6m | -0.226 | 0.795 | 0.80(0.11-4.12) |  | Non-convergence |  | 0.606 | | 0.094 | | 1.83(0.91-3.75) |
| *Defined as any combination of delusions of mind reading, thought broadcast, thought echo, thought insertion and thought withdrawal. | | | | | | | | | | | |
